# A Plant Growth-Promoting Microbial Soil Amendment Dynamically Alters the Strawberry Root Bacterial Microbiome

**DOI:** 10.1038/s41598-019-53623-2

**Published:** 2019-11-27

**Authors:** Siwen Deng, Heidi M.-L. Wipf, Grady Pierroz, Ted K. Raab, Rajnish Khanna, Devin Coleman-Derr

**Affiliations:** 1 Department of Plant and Microbial Biology, University of California, Berkeley, CA USA; 2 Plant Gene Expression Center, USDA-ARS, Albany, CA USA; 3 Carnegie Institution for Science, Department of Plant Biology, Stanford, CA USA; 4 i-Cultiver, Inc., 404 Clipper Cove Way, San Francisco, CA USA

**Keywords:** Microbiology, Plant sciences

## Abstract

Despite growing interest in utilizing microbial-based methods for improving crop growth, much work still remains in elucidating how beneficial plant-microbe associations are established, and what role soil amendments play in shaping these interactions. Here, we describe a set of experiments that test the effect of a commercially available soil amendment, VESTA, on the soil and strawberry (*Fragaria x ananassa* Monterey) root bacterial microbiome. The bacterial communities of the soil, rhizosphere, and root from amendment-treated and untreated fields were profiled at four time points across the strawberry growing season using 16S rRNA gene amplicon sequencing on the Illumina MiSeq platform. In all sample types, bacterial community composition and relative abundance were significantly altered with amendment application. Importantly, time point effects on composition are more pronounced in the root and rhizosphere, suggesting an interaction between plant development and treatment effect. Surprisingly, there was slight overlap between the taxa within the amendment and those enriched in plant and soil following treatment, suggesting that VESTA may act to rewire existing networks of organisms through an, as of yet, uncharacterized mechanism. These findings demonstrate that a commercial microbial soil amendment can impact the bacterial community structure of both roots and the surrounding environment.

## Introduction

Agriculture faces numerous abiotic and biotic challenges in optimizing crop production. Drought and excess salinity impact 45% and 19.5% of agricultural land, respectively^1–3^, and plant disease is one of the leading constraints of agricultural productivity^4,5^ that accounts for total annual yield losses of US$220 billion globally^6^. Achieving food security is further compounded by the effects of climate change and rapid population growth^7,8^. While preventative measures and early management of stresses are key to fortifying crop yield, current strategies often have calamitous ramifications and are unsustainable. Widely employed methods include the heavy application of exogenous fertilizers^9^, growth enhancers^10^, pesticides^11^, and soil sterilization methods, such as fumigation with methyl bromide (MeBr)^12^. While these treatments can be effective for managing stressors, they also can have dire consequences for human health, the environment, and long-term soil quality and health^13^. Adverse impacts of these methods include water, air, soil, and food contamination, increased incidences of cancer, the disruption of reproductive, neurological, and respiratory systems of mammals, birds, and fish, reductions in pollination and other important ecosystem services, ozone depletion, and eutrophication^14–19^. While the regulation of several widely-used chemicals has increased in recognition of these issues^20–22^, new approaches are needed to further support and improve crop performance.

One promising alternative is the use of plant growth-promoting microbes (PGPM). Recent work has shown that plants recruit specific microbial colonists via root and leaf metabolites, some of which are exuded into the soil and surrounding environment^23^. In exchange for these compounds, PGPM can promote plant growth through a variety of mechanisms, including increasing soil nutrient bioavailability, improving water acquisition, decreasing herbivore damage, and suppressing plant disease^24–27^. Along with the enhancement of plant productivity and yield, PGPM can allow for significant reductions in the application of chemical fertilizers and pesticides^28,29^. In light of these benefits, there is a growing number of microbial amendments being commercialized for various crops^30–33^.

There is ongoing research on how certain PGPM affect crop performance and the resident soil and plant-associated microorganisms^34,35^. Field inoculation of rhizobia strains enhanced populations of Alpha- and Gamma-proteobacteria, together with Firmicutes and Actinobacteria, in the bulk soil of common bean plants^36^. However, inoculation of Azospirillum strains showed no prominent effects on population structure of rhizobacterial communities in maize^37^. Yet, few studies have explored the effect of community-level microbial amendments on the native soil microbiome, the survival of applied microorganisms in the field, and the temporal community dynamics of host-associated epiphytes and endophytes following amendment application. Additional research is needed to understand how microbial-based methods can be employed for improving crop growth.

In this study, we explore the effect of a commercially available microbial soil amendment, VESTA, on the microbiome of strawberries, an important horticultural crop. Strawberries are valued at US$2.8 billion nationally^38^ and face a number of stress-related challenges^39^. Past work has shown that its growth can be regulated by microbial means^40–42^, and 16S rRNA and 18S ITS gene amplicon-based metagenomics approaches have identified microbes that potentially play a role in yield decline in strawberry^43^, where low abundances of beneficial bacteria and a nematode fungus, in addition to high levels of fungal root rot pathogens, have been implicated. Taken together, these recent findings suggest that manipulating the strawberry microbiome may be an effective way to increase plant growth.

VESTA is a fermented liquid product composed of a broad spectrum of microbes, fermentation by-products and organic acids (VESTA, SOBEC Corporation, Fowler, CA), and the direct effect of VESTA on microbial communities in the soil or plant host is unknown. Here, we investigate the effect of the amendment on soil physicochemistry, strawberry growth, and bacterial community composition, diversity, and function. We observe phenotypic differences in overall biomass and lateral root growth in amendment-treated strawberry plants. 16S rRNA gene amplicon-based Illumina sequencing revealed substantial changes in the strawberry microbiome following treatment with the amendment, but suggests that this effect is the result of the amendment modulating the compositional profile of existing root and soil microbes, rather than through replacement of the community with the product’s microorganisms. Shotgun sequencing of the amendment largely corroborated the community composition profile obtained through amplicon sequencing, and offers insight into its potential function. Together, these findings demonstrate that a commercial microbial soil amendment can alter the community structure of the strawberry root and surrounding environment.

## Methods

### Site description and amendment application

To investigate the effect of the microbial soil amendment on field-grown strawberries, soil properties, and bacterial communities associated with the host and soil, we designed an experiment that allowed for collection of amendment-treated and untreated control samples from a commercial strawberry farm in Guadalupe, CA (34.9716°N, 120.5718°W; Supplementary Information Fig. S1). The sandy loam soil of the strawberry operation is a mixture of sandstone-derived alluvium from the Coast Range and wind-borne materials^44^. Plugs of a day-neutral strawberry cultivar (*Fragaria x ananassa* Monterey) were transplanted to beds in two 12-acre plots, and the microbial soil amendment VESTA was applied to one of two neighboring plots in a two-block design, due to limitations of the commercial farm site’s irrigation system. Before the start of this study, the field utilized had been uniformly planted with broccoli (*Brassica oleracea* var. *italica*) the previous season and received identical watering and fertilizing treatments and crop rotations. The microbial soil amendment VESTA was fertigated via drip irrigation systems to the treated plot starting at seven days after planting, and the freshly prepared product was applied each month from January through August during the year of 2015. At the time of each application, 75 liters of VESTA was applied per acre. Water in the system was run for approximately 30 minutes in order to transfer the product into the strawberry root zone. Treated and control plants were fertilized similarly, with recommended practices for a growing season being 54, 14–27, and 54 kilograms per acre of nitrogen (N), phosphorus (P), and potassium (K)^45^, respectively, and fertilizer was applied monthly via drip irrigation lines. VESTA itself contributed minimally to overall levels of N, P, and K supplied to the plants at 150, 420, and 1635 grams per application, respectively (Supplementary Information Table S1). These levels are very low compared to standard fertilizer inputs, and even with the higher relative degree of supplementary K, it has been reported that up to 2/3-fold additional K above recommended annual dosage gave no apparent benefit to strawberry plant growth^45^.

### Sample collection and processing

Four replicate samples each of soil and whole root systems were collected from both treatments before (time point 1), during (time points 2 and 3) and after (time point 4) the fruit harvesting season, which were approximately two, three, four and seven months after planting. Soil samples were collected from the topsoil approximately 15 centimeters (cm) from each individual plant that was sampled, and whole root systems were collected by shoveling to a depth of approximately 20 cm and sent to the lab overnight with ice packs (Supplementary Information Fig. S1). Samples were stored at −80 °C until processing. The root endosphere and rhizosphere fractions were separated as detailed by Simmons *et al*.^46^. Whole roots were placed in epiphyte removal buffer (0.75% KH2PO4, 0.95% K2HPO4, 1% Triton X-100 in ddH2O; filter sterilized at 0.2 μM) and roots and rhizosphere were separated using a sonication method (pulses at 160 W for 30 seconds, separated by a 30 second pause for 10 minutes at 4 °C), followed by removal of roots into fresh tubes and two subsequent steps of rinsing with sterile water. The remaining rhizosphere fraction was pelleted by centrifugation at 300 rpm for 1 minute and the excess supernatant was removed. Both root endosphere and rhizosphere samples were then stored in new sterile epiphyte removal buffer at −80 °C until DNA extraction. An additional plant collection (n = 6 for each treatment group and time point) occured for measurement of root fresh weight at each time point. For these measurements, soil was first removed from root tissue by gentle rolling over a 4 mm mesh and a gentle rinse with DI water, and subsequent shaking to remove residual water and weighed to measure fresh weight. Roots then received a 2 hour incubation at 90 degrees Celsius, followed by a 55 degree Celsius treatment for 3 days, and roots were weighed again to calculate root dry weight. Root tissues were subsequently dried in an oven at 37 degrees Celsius for two weeks prior to measurement of dry weight. Water content of the roots was calculated using (RFW − RDW)/RFW.

### Soil chemistry analysis

For a broad survey of the amendment product’s influence on bulk soil chemistry, we utilized EDXRF for non-destructive analysis of soils (Supplementary Information Table S2a). Samples weighing 1.80 to 1.85 grams of the <2 mm sieved and dried soils were analyzed in a Spectro XEPOS HE spectrometer (AMETEK Inc.; Berwyn, Pennsylvania, USA). Each sample was interrogated at 4 spots on a 35 mm-diameter planchet, and the values averaged after normalizing to a Compton scattering background.

To standardize the chemical and physical tests and permit ease of handling, control and amendment treated soils were gently warmed from −8 °C to 3 °C overnight. Soil physical observations were performed using a <2 mm sieve to determine wet-cohesive strength, and physical manipulation was used to determine textural characteristics and average aggregate-size using oven-dried (55 °C for 48 to 72 hours). Two grams of soil solubilized in the saturated paste extract method was used in determining soil temperature-corrected pH with an Ag/AgCl electrode (Fisher Scientific), and electrical conductivity (EC_10_) with a conductivity probe Russell RL060-C (W.W. Grainger, Inc., Lake Forest, IL, USA; Supplementary Information Table S2b)^47^. The <2 mm sieved fraction of two soil samples per treatment were analyzed first for soil carbon and nitrogen using a Carlo Erba 1500NA elemental analyzer (Carlo Erba; Milan, Italy). Separate subsamples were extracted^48^ with 2 M KCl to determine nitrate and ammonium levels with a WESCOR SmartChem 200 discrete analyzer (Unity Scientific; Milford, MA), and exchangeable cations from soil estimated using 1 M (pH = 7.0) ammonium acetate extractions, followed by inductively coupled plasma-optical emission spectrometry (ICP-OES).

### Strawberry plant and product nutrient analysis

The edible portions, strawberry aggregates of achenes, rely on the concerted efforts of the root zone and attendant microbiota to mine available nutrients from the soil that are then transported to the fruit. For all below ground tissue harvested at time points 1–4, Ca, Mg, K, Na, P, S, and micronutrients were analyzed with ICP-OES (Supplementary Information Table S3a). Tissue was rinsed with distilled water, dried slowly at 55 °C (48–72 h), and ground to flour consistency using a reciprocating ball-mill (SPEX Inc.; Metuchen, NJ). Powder was then digested using an overnight soak in concentrated HNO_3_ at room temperature, followed by heating to 180–210 °C in a programmable heating block in 2:1 (v/v) HNO_3_:HClO_4_, as described in^49^. All values are expressed on a mg/kg oven-dry basis, utilizing subsamples dried to constant weight at 105 °C. Foliar tissues were only compared at time point 4, the final harvest, in order to not interfere with commercial fruit harvest. Composited from 5 plants spatially-dispersed in the control field and the amendment-treated field each, leaves were dried and ball-milled as described above. Energy-dispersive X-ray fluorescence (EDXRF) was then used to analyze a suite of elements similar to that for roots (Supplementary Information Table S3b). Yield was not quantified at the time of this study. The nutrient profile of the liquid product VESTA was assessed by D&D Agricultural Laboratory, Inc. (Fresno, CA) by means of a pH analyzer, electrical conductivity meter for soluble salts, FP-528 (Leco Corporation, St. Joseph, MI) for total nitrogen, and Optima 8000 ICP-OES (PerkinElmer, Inc., Waltham, MA) for all other parameters (Supplementary Information Table S1). Due to limitations in the quantity of tissue available per sample, and differences in minimum tissue amounts necessary for different test, replication levels vary from test to test across the study.

### DNA extraction and PCR amplification

Soil and rhizosphere DNA was extracted using extraction kits (MoBio PowerSoil DNA Isolation Kit, MoBio Inc., Carlsbad, CA) following the manufacturer’s protocol. For root endosphere samples, roots were homogenized using mortar and pestle in liquid nitrogen and DNA was extracted using a modified CTAB DNA extraction procedure^50^. Due to high humic substances in root endosphere DNA, which potentially inhibit PCR reaction, we performed a cleanup step after DNA extraction using a modified MoBio PowerSoil kit protocol supplied by the manufacturer. We previously tested that DNA extraction method had no significant effect on the DNA preparation method^50^. We amplified V3-V4 region of 16S ribosomal gene using a dual-indexed 16s rRNA Illumina iTags primer (341 F (5′-CCTACGGGNBGCASCAG-3′) and 785 R (5′-GACTACNVGGGTATCTAATCC-3′) as described in^51^ using 5-Prime Hot Master Mix (catalog No. 2200410). After DNA extraction, DNA samples were diluted to 5 ng/ μl and randomized in 96-well plates. Water blanks were included on each 96-well plate as negative controls. PNA clamps were used to minimize host-derived amplicons from both chloroplast and mitochondrial 16S rRNA gene sequences^52^. Reactions included 11.12 μL DNase-free sterile H20, 0.4 μg BSA, 10.0 μL 5-Prime Hot Master Mix, and 2 μL template, and 0.75 μM of chloroplast and mitochondria PNAs. PCR reactions were performed in triplicate in three thermocyclers (to account for possible thermocycler bias) with the following conditions: initial 3 min cycle at 94 °C, then 30 cycles of 45 seconds at 94 °C, 10 sec at 78 °C, 1 min at 50 °C, and 1.5 min at 72 °C, followed by a final cycle of 10 min at 72 °C. Triplicates were then pooled (128 samples per library) and DNA concentration for each sample was quantified using Qubit reader. Pools of amplicons were constructed using 100 ng for each PCR product. Before submitting for sequencing, pooled samples were cleaned up with 1.0X volume Agencourt AMPureXP (Beckman-Coulter, West Sacramento, CA) beads according to the manufacturer’s directions, except for the modifications of using 1.0X rather than 1.6X volume beads per sample, dispensing 1500 μL 70% EtOH to each well rather than 200 μL, and eluting in 100 μL DNase-free H20 rather than 40 μL. An aliquot of the pooled amplicons was diluted to 10 nM in 30 μL total volume before submitting to the QB3 Vincent J. Coates Genomics Sequencing Laboratory facility at the University of California, Berkeley for sequencing using Illumina Miseq. 300 bp pair-end with v3 chemistry. Sequences were returned demultiplexed and with adaptors removed.

### Amplicon sequence processing, OTU classification and taxonomic assignment

Sequencing data was analyzed using the iTagger pipeline developed by the U.S. Department of Energy’s Joint Genome Institute^53^. This pipeline wraps several packages for the filtering, merging, clustering and taxonomy assignment, including CUTADAPT, FLASH, USEARCH, and RDP^54–57^. In brief, after filtering 28,581,170 16S rRNA raw reads for known contaminants (Illumina adapter sequence and PhiX), primer sequences were trimmed from the 5′ ends of both forward and reverse reads. Low-quality bases were trimmed from the 3′ ends prior to assembly of forward and reverse reads with FLASH^55^. The remaining 18,717,158 high-quality merged reads were clustered with simultaneous chimera removal using UPARSE^58^. After clustering, 11,204,438 read counts mapped to 13, 545 operational taxonomic units (OTUs) at 97% identity. The resulting reads produced on average approximately 72,298, 88,493 and 76,070 reads per sample for soils, rhizospheres, and roots respectively. Taxonomies were assigned to each OTU using the RDP Naïve Bayesian Classifier^57^ with custom reference databases. For the 16S rRNA V3-V4 data, this database was compiled from the May 2013 version of the GreenGenes 16S database^59^, the Silva 16S database^60^ and additional manually curated 16S rRNA sequences, trimmed to the V3-V4 region. After taxonomies were assigned to each OTU, we discarded 1) all OTUs that were not assigned a Kingdom level RDP classification score of at least 0.5, 2) all OTUs that were not assigned to Kingdom Bacteria. To remove low abundance OTUs that are in many cases artifacts generated through the sequencing process, we removed OTUs without at least 5 reads in at least 5 samples. We also removed samples have less than 10,000 reads, which yielded 3,387 high-abundance OTUs (respectively) for downstream analyses. These thresholds were found to be suitable using technical replicates in a dataset published previously^61^. To account for differences in sequencing read depth across samples, all samples were rarefied to 16,542 reads per sample for specific analyses, or alternatively, by dividing the reads per OTU in a sample by the sum of usable reads in that sample, resulting in a table of relative abundance frequencies; OTUs which were reduced to less than one read per OTU after rarefaction were discarded to yield 2,464,758 measurable, rarefied reads for downstream analysis. The raw sequencing reads for this project will be deposited in the NCBI Short Read Archive SUB3636449.

### Shotgun metagenomic sequence processing and analysis of amendment product

In order to assess the metagenomic composition of the VESTA product itself, three 50 mL aliquots were made from the same batch of VESTA and treated as technical replicates. To extract genomic DNA, the 50 mL aliquots of homogenized product were centrifuged at 10,000 rpm for 20 minutes. The supernatant was discarded, and the pellet was then re-homogenized. Of the resulting sediment, 0.250 grams were processed according to manufacturer instructions with the PowerSoil DNA Isolation Kit (MO BIO Laboratories, Inc., Carlsbad, CA, USA). DNA was sheared to 300 bp using a Covaris Focused-ultrasonicator (Covaris, Inc., Woburn, MA, USA), and then used to generate libraries with the Kapa LTP Library Preparation Kit (Kapa Biosystems, Inc., Wilmington, MA) and adapters and unique barcodes provided by the QB3-Berkeley facility to allow for multiplexing. Sequencing was performed at QB3-Berkeley on the Illumina HiSeq. 2500 system with 150 bp paired-end reads. De-multiplexing was performed by the California Institute for Quantitative Biosciences (QB3-Berkeley) Functional Genomics Laboratory. We received sequences for 266 million reads, or an average of 88.5 ± 4.1 million reads per technical replicate, for a total of 45.5 billion bases. Raw reads were uploaded to Metagenomics RAST server (MG-RAST) for paired-end joining, quality control, and taxonomic and functional annotation^62^. The raw metagenome sequencing reads for this project will be deposited in the NCBI Short Read Archive SUB3688024. After quality control, one of the three technical replicates was revealed to be of much lower quality and was discarded. The two replicates we chose to retain accounted for a total of 172 million reads, of which 21.1 million (12.3%) failed quality control, 11.4 million (6.63%) could not be identified, and 139 million (81.1%) were assigned functional and/or taxonomic annotations according to default parameters (alignment length cutoff = 15 bp, e-value cutoff = e-5, percent identity cutoff = 60%) using RefSeq database^63^. Of those sequences which could be identified, 329,113 sequences (0.24%) contain ribosomal RNA genes, 63.6 million sequences (45.7%) contain predicted proteins with known functions, and 75.2 million sequences (54.0%) contain predicted proteins with unknown function. Over 97% of all reads were determined to belong to bacteria, compared to only 0.12% of reads being assigned fungal identity. For this reason, we decided to focus solely on the bacterial sequences in this study. Functional annotations according to the SEED subsystem database^64^ are presented in this publication.

### Statistical analysis

RStudio (version 1.0.136; RStudio Team) was utilized for all statistical analyses with the packages phyloseq.^65^ and vegan^66^. For plant phenotype data, scatter plots were generated using ggplot2, and Analysis of Variance (ANOVA) was performed with function aov. For the Alpha diversity measurement, Shannon Index of diversity and observed OTUs were calculated with the estimate_richness function in the R package phyloseq. ANOVAs were performed with function aov for Sample Type, Treatment, and Time Point. A Tukey’s Post Hoc test was performed using function TukeyHSD in the stats package and with HSD.test in the package agricolea to test which levels were significantly different from one another. Beta diversity was measured using Bray-Curtis distances and UniFrac distance with function ordinate in the R package phyloseq. For UniFrac distances, trees were built with default parameters using FastTree^67^ with an alignment constructed in Muscle^68^. Permutation multivariate analysis of variance analyses (PERMANOVA) were performed with the Adonis function in the R package Vegan using 999 permutations and the Bray-Curtis distances as inputs. Canonical Analysis of Principal Coordinates (CAPs) was performed for subsets of the data with each sample type and time point to determine the percent variance explained by treatment, time point and replicate, or treatment, sample type, and replicate, respectively, using the capscale function in the R package vegan^66^. The non-parametric Kruskal-Wallis test in R was used to compare Shannon indices and class-level relative abundances between treated and untreated within each time point and sample type. Indicator species analyses run on root samples to determine genera that were enriched for either control or amendment treatments were performed using R package indicspecies^69^, with p-values < 0.01 based on permutation tests run with 999 permutations. All scripts used can be found at a public repository on github (https://github.com/siwendeng/Strawberry-Microbiome).

## Results

### The microbial soil amendment VESTA enhances strawberry yield and alters soil chemistry

**Figure 1:**
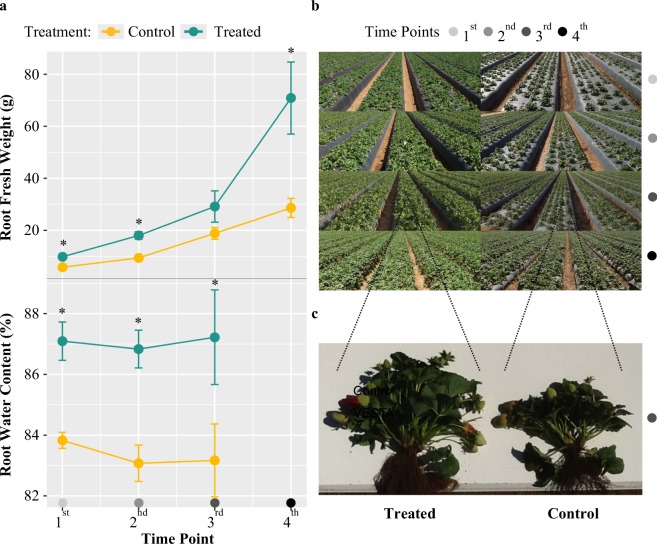
Treatment effect on strawberry root growth. Amendment-treated strawberry plants display evidence of root growth promotion, as compared to controls. (**a**) Line plot illustrating the average trend of 5 to 6 replicates per treatment per time point for root fresh weight (RFW) in grams and percentage Root Water Content (RWC) in amendment-treated (indicated in blue) and control (yellow) strawberry plants across the four sampling time points. Data shown are mean ± SE. ‘*’ indicates p-value < 0.01 (**b**) Photographs of amendment-treated and control strawberry fields across four time points in upper panel. Four time points correspond to sampling dates of March 18 (light grey circle), April 8 (grey circle), May 13 (dark grey circle), and August 24 (black circle) in 2015. (**c**) Two representative plants from treated and control fields from the 3rd sampling time point in lower panel. Amendment-treated strawberry plant displays enhanced root proliferation at the crown and greater aboveground shoot biomass.

Roots from strawberries grown in amendment-treated fields displayed enhanced growth, as evidenced by significantly greater fresh weight across time points in comparison to control roots (Fig. 1a). This indicates that amendment-treated samples may differ from control in their greater ability to absorb water from the surrounding soil. Visual inspection revealed that this effect was likely from enhanced root-proliferation at the crown and greater aboveground shoot biomass (Fig. 1b). Additionally, amendment-treated roots only had significantly greater dry weight in the first and second time points out of the three time points that this trait was measured (Supplementary Information Fig. S2). Lastly, water content of amendment-treated roots was uniformly higher than in the control plants (Fig. 1a), which can impact photosynthesis, plant performance, and actualized growth potential^70^.

In comparison to control soils, soils treated with the soil amendment had a higher wet-cohesive strength, greater mass plant-derived materials, and increased average soil particle aggregate-size. Electrical conductivity (EC_10_) was also higher in amendment-treated soil compared to control soils (0.242 dS/m vs. 0.184 dS/m; Supplementary Information Table S2a). Comparing nitrate levels, amendment-treated soil was composed of distinctly less nitrate than the controls (Supplementary Information Table S3a). Total nitrogen of treated soils was 0.188% dry weight, while for controls it was 0.177% dry weight; for total carbon content, treated soils contained 0.385% dry weight while control soils contained 0.249% dry weight (Supplementary Information Table S3a). Based on energy-dispersive X-ray fluorescence (EDXRF), overall micronutrients were lower in the treated soils than controls (Supplementary Information Table S3b). In comparison to control plants, the roots of plants treated with the amendment product also had significantly higher phosphorus levels (2315 µg/g vs. 1747 µg/g of tissue) and significantly lower levels of aluminum, iron, and molybdenum; foliar tissue collected from treated plants at time point four showed generally greater levels of micronutrients than those collected from control plants (Supplementary Information Table S3). Taken together, these results demonstrate that amendment application has a significant effect not only on root growth, but on soil physiochemistry as well. However, with the constraints of a two block design, we acknowledge that there is an unknown degree of spatial influence on soil physiochemistry, as well as on bacterial community assembly, and additional studies utilizing a fully randomized block design are needed to confirm these reported effects of the microbial soil amendment.

### Bacterial community profiling of amendment-treated and control soil, rhizosphere, and roots

To allow for a direct comparison of microbes present in the amendment and microbes observed in our treated samples, samples of the product were also processed and sequenced using the same protocol. After demultiplexing, quality control, clustering based on 97% similarity threshold, and assigning taxonomies, 13,545 OTUs were generated. To remove underrepresented sequences which often represent sequencing artifacts^53^, we filtered these OTUs by removing OTUs not seen more than 5 times in at least 3% of the samples (n = 5). After filtering, we resampled the OTU table to normalize for differences in read count abundance between samples to a common read depth of 16,542 reads per sample. The resulting dataset of 3,387 OTUs was used in the downstream statistical analyses.

### Bacterial diversity is affected by amendment treatment

**Figure 2:**
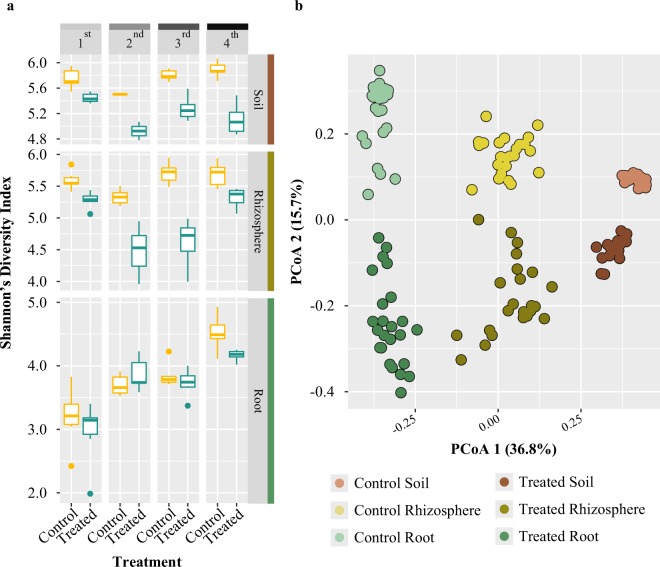
Treatment effect on strawberry root microbiome. 16S rRNA bacterial profiling indicates treatment impacts Shannon’s Diversity most distinctly in soil and rhizosphere samples and that samples distinctly cluster by sample type and treatment. (**a**) Box and whisker plots representing Shannon’s Diversity indices for all samples based on Bray-Curtis distances in amendment-treated (blue) and control (yellow) samples for each sample type and at each of the four sampling time points; soil (upper panel), rhizosphere (middle panel), and root (lower panel) indicate that amendment treatment correlates with a decrease in the within-sample diversity in soil and rhizosphere samples, but not in roots. The horizontal line within each box represents the median index value, and the bottom and top edges of each box indicate the 25th and 75th percentiles, respectively. Individual points are outliers. (**b**) Principal coordinate analysis (PCoA) plot for all samples using Bray-Curtis distances indicates that the largest source of variation between microbial communities is sample type (PCo 1, 36.8%) and the second largest source of variation is treatment (PCo 2, 15.7%). Both control soil (light brown circles), rhizosphere (light yellow), and root (light green) samples and amendment-treated soil (dark brown), rhizosphere (dark yellow), and root (dark green) samples cluster together within their respective treatment and sample types.

To investigate the impact of amendment application on bacterial community composition in the root, rhizosphere, and surrounding soil, we performed 16S rRNA amplicon sequencing on the Illumina MiSeq platform using custom V3-V4 dual-indexed primers^50,51^. We hypothesized that the addition of a complex microbial amendment would increase overall microbial diversity within the soil and root microbiomes. Rarefaction curves were used to estimate species richness as a function of sequencing depth and suggest that saturation in sequencing was achieved, as curves began to plateau or reached their asymptote for all sample types by treatment (Supplementary Information Fig. S3). Unexpectedly, we found that total community diversity, as measured by Shannon’s Diversity, decreased in treated samples as compared to control samples, for both soil and rhizosphere sample types at every time point (Fig. 2a; Supplementary Information Table S4a). By contrast, Shannon’s Diversity in the root in time points 1, 2, and 3 was not significantly different between treated and control samples (Fig. 2a; Supplementary Information Table S5). Additionally, there is a significant effect of time point on Shannon’s Diversity (F value = 21.555, p < 0.001; Supplementary Information Table S4b). From time point 1 to 4, Shannon’s Diversity decreases in treated soil samples (Fig. 2a). In the rhizosphere, Shannon’s Diversity decreases from the 1st to the 3rd time point in treated samples; however, in the 4th, it increases to a level more similar to the Shannon’s diversity observed in the control samples (Fig. 2a). In the root, Shannon’s diversity in treated samples is similar to levels in control samples, increasing from time point 1 to 2, and then gradually decreases again for all successive time points (Fig. 2a). Finally, the mean number of observed species was found to be lower in amendment-treated samples than control samples in all sample types and time points (Supplementary Information Fig. S4a). Taken together, these results suggest that amendment application alters root-associated microbial communities and is correlated with an overall decrease in bacterial diversity in the treated area, but that this effect is mitigated in some way within the plant host.

### Amendment application is correlated with shifts in bacterial community composition

**Figure 3:**
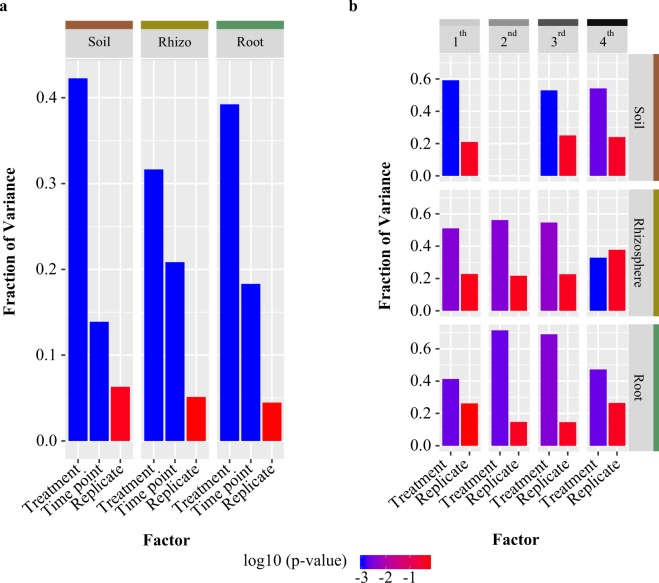
Treatment type explains the greatest bacterial community variance for each sample type and time point. (**a**) Bar plots displaying fraction of variance explained by treatment, time point and replicate, as determined by canonical analysis of principal coordinates (CAPS) using Bray-Curtis distances. The y-axis indicates the fraction of variance explained by each factor, and the shade of the bar indicates the level of significance (p > 0.01 is in light grey; p < 0.001 is in dark grey). (**b**) Bar plots displaying fraction of variance determined by CAPS using Bray-Curtis distances and performed separately for each time point (1^st^, 2^nd^, 3^rd^, and 4^th^) on soil (shown in the upper panel), rhizosphere (middle panel), and root (lower panel) samples. The fraction of variation is indicated by the y-axis, and the shade of the bar indicates the level of significance.

To test which factors in our experimental design contribute to differences in the beta diversity of bacterial communities associated with the soil, rhizosphere, and root, PERMANOVA analyses were performed independently on each sample type using Bray-Curtis distances. These results indicated that the effect of amendment application on community composition was larger in soil than in the root and rhizosphere samples (Supplementary Information Table S6). These results also revealed that time point influenced community composition (Supplementary Information Fig. S4b; Table S6), and that this effect was least pronounced in soil samples as compared to root and rhizosphere. To further visualize whether amendment treatment influenced bacterial community composition in the soil, rhizosphere, and root, we conducted principal coordinate analysis (PCoA) for all sequenced samples using both Bray-Curtis and UniFrac distances (Fig. 2b; Supplementary Information Fig. S4b). PCoA using the Bray-Curtis dissimilarities (Fig. 2b) displayed strong clustering of samples by sample type along the first PCoA axis, which explains 36.8% of the variance. Additionally, we observed that samples cluster by treatment type along the second PCoA axis, which explains 15.7% of the variance. PCoA based on weighted UniFrac distances (Supplementary Information Fig. S4b) revealed similar trends. The result that the effect of time point was least pronounced in soil samples is observable in the PCoA plot, where for root and rhizospheres, amendment-treated samples belonging to the fourth time point were found to cluster closer to the untreated samples (Supplementary Information Fig. S4b). Notably, samples taken at time point four were treated more than a month prior to sampling, whereas samples collected at all other time points had been treated one or two weeks prior to sampling. This suggests that in the interim between treatments, the soil and root microbiomes in the treated field may drift back towards a compositional profile more similar to that of control fields. To determine the percent of variance explained by the factors shown to be significant by PERMANOVA (time and treatment, and their interaction; Supplementary Information Table S6), we next performed a canonical analysis of principal coordinates (CAPS) using the Bray–Curtis distance independently on each sample type (Supplementary Information Table S7a). Results were very similar to those determined by PERMANOVA, where treatment explained the largest proportion of variance across all sample types (42%, 32%, and 39% in the soil, rhizosphere, and root, respectively; Fig. 3a). The percent variance explained by time point is largest in the rhizosphere (21%), followed by the root (18%) and smallest in the soil (14%; Fig. 3a). A CAPS analysis performed separately for each time point and constrained for treatment type showed that time point effects on community composition are more pronounced in the root and rhizosphere (Fig. 3b; Supplementary Information Table S7b), which suggests that the extent to which the amendment impacts bacterial communities varies across plant development and may be influenced by shifts in microbial recruitment and community maintenance via exudation rates and profiles. In addition, treatment explains less variation at time point four in root and rhizosphere, but not in soil (Fig. 3b; Supplementary Information Table S7b). Taken together, these results suggest that treatment with the amendment has an effect on bacterial community composition not only in the soil, where it is applied, but also within and on the plant root.

### Amendment treatment is correlated with increases in *Betaproteobacteria*

**Figure 4:**
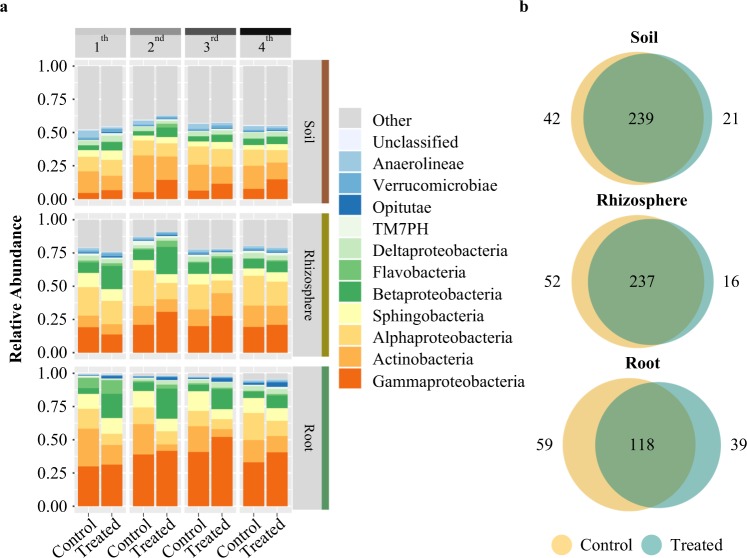
Class-level relative abundance of bacterial communities indicate distinct soil, rhizosphere, and root profiles. (**a**) Relative abundances bar graphs for the top 12 most abundant bacterial classes for amendment-treated and control samples in soil (upper panel), rhizosphere (middle panel), and root (lower panel) across four time points (1^st^, 2^nd^, 3^rd^, 4^th^). (**b**) Venn-diagrams displaying numbers of OTUs shared and distinct between amendment-treated (blue) and control (yellow) samples in soil (upper panel), rhizosphere (middle panel), and root (lower panel) compartments.

Amendment treatment was correlated with changes in relative abundance at different taxonomic levels within all sample types (Fig. 4a). Changes in the roots were characterized at the class level by a significant increase in *Betaproteobacteria* (P < 0.001, Chi square = 33.93) and a concomitant decrease in the abundance of *Actinobacteria (*P < 0.001, Chi square = 23.38). An indicator species analysis was used to identify at higher taxonomic resolution the individual bacterial genera with enrichment or depletion patterns in roots treated with the amendment as compared to controls. These analyses revealed that genera from the families *Alteromonadaceae*, *Comamonadaceae*, *Burkholderiales*, which are known to contain species with beneficial properties to plants^71^, were all observed to be more abundant in amendment-treated roots. In addition, several families and genera of *Betaproteobacteria* (*Ramlibacter, Rhodocyclales, Methylophilaceae, Methylotenera, Acidovorax, Comamondaceae*) that were significantly enriched in amendment-treated samples versus control samples have the capabilities of nitrogen fixation^72–74^, denitrification^75,76^, and sulfur cycling^77^ (Supplementary Information Table S8). However, it is important to note that the specific OTUs observed in our study that are represented by these indicator lineages may or may not possess these properties noted in the literature; additional work in the future to reveal the functional capacities of individual isolates through isolate sequencing and phenotyping will help to resolve this knowledge gap.

Based on our observation that the amendment has an overall larger effect on soil microbiome composition than roots, we hypothesized that a larger number of OTUs would be shared between treated and control samples in roots, as compared to soils. However, we observed that the percentage of differentially present OTUs between amendment-treated and control samples is greatest in roots, where 594 (27.2%) and 396 (18.2%) OTUs are distinct to control and amendment-treated, respectively, with only 54.6% of the OTUs held in common (Fig. 4b). By comparison, 2399 (79.0%) and 2378 (77.6%) OTUs are shared across soil and rhizosphere samples, respectively.

### Community compositional changes associated with VESTA treatment are not driven by an increase in abundance of VESTA organisms

To investigate whether the changes in community composition in the soil, root and rhizosphere following inoculation with the amendment product are due to increased colonization by microorganisms present within VESTA product, we first sought to characterize the microbial diversity, community composition, and compositional stability within the product. As the product VESTA is created by first mixing two raw pre-products, BHF-10 and SOBEC, we collected initial samples of both pre-products, and weekly samples of VESTA for 14 weeks. Bray-Curtis distance of pairwise treated time point samples also showed that the product changes significantly over time (Supplementary Information Fig. S5). As amendment application is typically administered using product prepared within one to two weeks, we considered the composition of VESTA samples after one and two weeks after the mixing of the pre-products as representative of the VESTA community applied in our strawberry field study for downstream analysis. To help eliminate potential taxonomic bias introduced by our choice of 16S rRNA primers used in our study, a shotgun metagenomic analysis of the VESTA product was also performed. Both shotgun and 16S datasets revealed a community composition of roughly 500 organisms (Supplementary Information Fig. S6a) that was largely comprised of the genera *Mycobacterium*, *Caulobacter*, *Novosphingobium*, *Bacillus*, *Flavobacterium*, and *Pseudomonas*, many of which are reported to contain strains with plant growth-promoting characteristics^71,78–80^. Due to the unexpectedly complex nature of the VESTA community, an investigation into the functional capacity of the product provided limited information on the product’s potential relevance for plant growth-promoting capabilities; however, we did observe an abundance of genes related to microbial stress responses, including dormancy, sporulation, and secondary metabolism, as well as phosphorus, sulfur, nitrogen, iron, and potassium metabolism, which could play a role in improving nutrient acquisition for the host plant (Supplementary Information Fig. S6b)^81^; however, these types of functions can be expected to be observable in a wide variety of microbial ecosystems, and additional functional analyses and validation experiments will be necessary to test these hypotheses.

**Figure 5:**
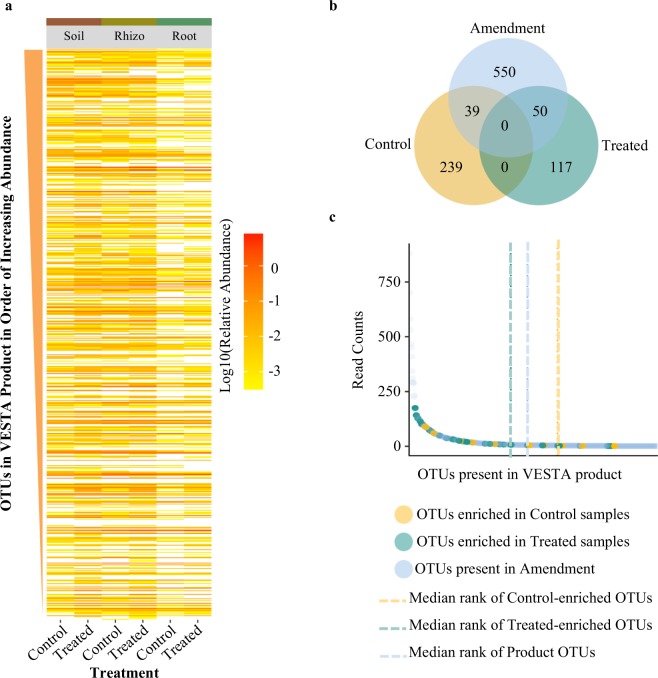
OTUs in the product are not highly enriched in treated or control samples. (**a**) Heatmap displaying degree of enrichment by log10 relative abundance in treated and control soil, rhizosphere, and root samples for the 639 OTUs present in the product. Product OTUs were sorted according to their relative abundance from top (highest) to the bottom (lowest). Darker shades of color indicate higher relative abundance within the samples, whereas lighter shades indicate lower relative abundance. Columns for amendment-treated soils, rhizospheres, and roots generally are darker, indicating greater enrichment in product OTUs than controls, but there was no significant enrichment. (**b**) Venn-Diagram displaying the OTUs shared and distinct to the product and those uniquely enriched in the amendment-treated or control roots, as determined with indicator species analysis. OTUs uniquely enriched in control (yellow) and treated (green) samples were compared with those present in the product (light blue). More OTUs from the product were distinctly shared with treated samples (50 vs. the 39 OTUs shared between the product and controls), and treated samples shared a larger proportion of total OTUs (30% or 50 of 167 OTUs total) with the product than controls (14% or 39 of 278 OTUs total). (**c**) All OTUs present in the product were ranked by their relative abundance, with the y-axis representing the average read count for each product OTU. The median rank for OTUs which showed enrichment in the control (yellow circles; n = 39) or treated roots (green circles; n = 50) was calculated and is displayed with yellow and green dashed lines, respectively; the median rank for all OTUs in the product with no enrichment (light blue circles; n = 550) is shown by the blue dashed line. This demonstrates that the median rank abundance in the product was roughly 1.5x higher in treated roots than that for OTUs enriched in controls.

We next explored the overlap between OTUs present in the VESTA product with all those uniquely enriched in amendment-treated and control samples across the three different sample types. Surprisingly, we did not observe a large enrichment in abundance of the majority of the OTUs belonging to the product within treated versus control samples in any compartment (Fig. 5a; Supplementary Information Fig. S7). However, a comparison of the lists of OTUs uniquely enriched in control and treated samples across all sample types as determined by indicator species analysis with the list of OTUs present in the product revealed that a larger proportion (30% or 39 OTUs) were members of the product in the treated samples than OTUs in the controls (14% or 19 OTUs; Fig. 5b). Additionally, the mean rank abundance within the product for all OTUs uniquely enriched in treated samples was roughly 1.5 fold higher than that for OTUs enriched in the control samples (Fig. 5c). Of the 639 OTUs present in the product, 436 (68.2%), 426 (66.7%), and 327 (51.2%) were observed to also be present within the amendment-treated soil, rhizosphere, and root compartments, respectively (Supplementary Information Fig. S7). By comparison, 396 (62%), 413 (65%), and 288 (45%) OTUs from the product were observed to also be present in the control soil, rhizosphere, and root samples, respectively.

Taken together, these results demonstrate that amendment treatment leads to a broad restructuring of the root microbiome, and that while treatment results in significant changes in microbial community composition, increases in the abundance of the microbes present in the product itself are not the primary driver of this difference.

## Discussion

Our 16S rRNA gene amplicon-based metagenomic analysis of soil, rhizosphere and root samples treated with the amendment product reveals that bacterial communities can be significantly altered by microbial amendment application. Unexpectedly, application of the amendment was associated with decreases in soil and rhizosphere Shannon Diversity. Similar results have been observed in previous studies; functional and bacterial and fungal diversity was shown to decrease post amendment application^82^, and the shift was observed to be dependent on specific parameters, including soil type, soil pore size (15–20 µm), and year^83,84^. However, other studies reveal that inoculation with certain soil amendments can instead increase or not impact soil and rhizosphere alpha diversity, including application of biochar^42,85,86^, vermicompost^87^, a heavy metal immobilizer amendment^88^, and bacterial inoculations^89^. One explanation for these discrepancies in effects may be the inherent complexity of each inoculant, and VESTA represents a more complex microbial system as compared with many of these other treatments. More generally, we expect that changes in microbial diversity in response to amendment application are likely dependent on a wide variety of factors, including edaphic and abiotic parameters of the environment being amended, but also the individual genetic and phenotypic characteristics of the microbes present in the amendment^90,91^. Additional studies in controlled settings that allow for additional environmental factors to be tested within the experimental design would further our understandings of the role of amendment application on soil and plant-associated microbial diversity.

As a pre-existing community of microorganisms, VESTA differs from many commercially available products in that its constituents already exist within the bounds of an established nutrient exchange system. The absence of these types of supporting relationships is often cited as the reason many single microbe inoculants that show promise in greenhouse studies fail to produce significant effects when tested in more complex field-based trials. While it has been observed that successful establishment of introduced microbes occurs less readily in diverse soils^92,93^, and some research also indicates that rhizosphere microbiomes are highly buffered against microbial invaders^94^, we anticipated that the product’s diversity would allow for increased colonization of its constituents within the root and rhizosphere. Surprisingly, we observed that the changes in community composition following treatment with the amendment were not primarily shifts in the abundance of bacterial organisms present in the product, but instead shifts in bacteria native to the environment. This suggests that the amendment is instead acting through an unknown mechanism to rewire the abundance patterns of existing soil and root microbes. As precedent for this, past research has found that bacterial inoculants on seeds are correlated with improved plant growth due to the stimulation of native microflora^95^.

One of the primary shifts we observed across all sample types was an increase in the relative abundance of *Betaproteobacteria* with amendment application. *Betaproteobacteria* are Gram-negative aerobic or facultative bacteria that are comprised of chemolithotrophs and phototrophs, and they have a broad range of metabolic capabilities, including the ability to fix nitrogen^96^. Increases in this class suggest treatment could hypothetically increase nutrient bioavailability for both the plant and the surrounding microbial communities. With an indicator species analysis, family and genera under the phylum of *Betaproteobacteria* that are highly enriched under amendment treatment have been ascribed the functions of denitrification and sulfur cycling; this may partly explain the reduced nitrate in amendment-treated soils, as compared to control soils. Reduced soil nitrate levels may also be partially explained by how larger plants would require additional nitrogen^97^. Another primary shift we found with amendment application was a decrease in abundances of *Actinobacteria*, particularly in amendment-treated roots. As two recent studies have demonstrated that water stress in crop roots systems is correlated with an increase in the abundance of many Actinobacterial lineages^50,98^, the observed decrease in Actinobacteria may be a result of the observed increase in water uptake in amendment-treated plants. Additional studies in other field sites will reveal which of these observed changes are typical for the application of the amendment, and which are specific to this experimental environment.

There are several mechanisms by which treatment with the amendment may be enacting community changes. Microbes in the product may outcompete a few, select native microbes and eliminate existing community hubs by means of greater resource use efficiency and metabolic potential. Amendment application may also cause organisms from the amendment and/or native microbes to produce new or higher levels of antimicrobials that affect overall diversity^99^. Metabolites produced initially from VESTA microbes may also directly impact plant growth, which then may cause indirect stimulation of a different community by influencing subsequent root growth and exudate release. In addition, the amendment product may directly contribute fungi or other microfauna that may then influence bacterial communities. While currently unknown, the specific mechanisms through which the amendment acts to modulate community structure, and whether this mechanism is also responsible for the observed growth promoting traits, will require further experimentation. VESTA has been utilized by commercial farmers in the past for its ability to improve plant disease resistance, growth, and yield. While the observed increase in plant biomass with amendment application may correlate with increased yield^100^, we did not measure yield in this study, and additional testing is needed in order to determine how soil treatment directly relates to microbial and genetic signatures of soil fertility, plant growth, and - in particular - yield and fruit quality (such as flavor, resistance to rot, and nutritional profile), as well as to overall shifts in microbial abundances and the fungal communities associated with the plant and soil. Furthermore, although this study was conducted under agriculturally relevant field conditions, and in a pair of plots which had received identical treatment in prior years, the two-block design used in this study limits our ability to draw conclusions regarding the statistical significance of the effect of VESTA in shaping the root microbiome; future work using additional replication and randomization, and in additional soil environments, will be useful for determining how generalizable these findings are.

Finally, we observed that many of the shifts associated with amendment application are dependent on time. In the final time point, the treated samples are more similar to control samples for all sample types. Importantly, in the fourth time point, samples were collected nearly one month after the most recent amendment application, in contrast to the other time points, in which the amendment had been applied two weeks prior to collection. This result is typical for studies conducted on the introduction of single organisms into complex soil systems. Several studies have found the general reduction of levels of individual inoculants introduced to soils^101–104^, finding as in little as a week there was more than a 99% reduction in abundance. Another study with pathogenic *Pseudomonas aeruginosa* showed a decline to below detectable levels after 3–5 weeks post introduction under non-sterile microcosms, while the population was maintained at high abundance under sterilized microcosms^105^. Our data suggest that even in cases where amendments represent complex communities of microorganisms, soil and root communities both have a resilience that will lean towards eventual recapitulation of the native state following microbial inoculation. This has important implications for the successful use of such products in commercial agriculture, and suggests that repeated applications may be beneficial for the persistence of the plant growth promoting agents.

Our 16S rRNA gene amplicon-based metagenomic analysis of soil, rhizosphere and root samples treated with the amendment product reveals that bacterial communities can be significantly altered by microbial amendment application. To our knowledge, this is the first reported instance of a microbial and nutrient-based soil amendment causing shifts in the bacterial communities present in the rhizosphere and root endosphere of a crop species. We find that the prescribed concentration of the product is effective in inducing changes in local bacterial communities, which may contribute to the root growth-promotion observed under treatment with the amendment.

## Supplementary information

Supplementary Information

## Data Availability

The datasets generated during the study are available in the NCBI repositories, SUB3636449 and SUB3688024.
