# Supplementary material for: A Plant Growth-Promoting Microbial Soil Amendment Dynamically Alters the Strawberry Root Bacterial Microbiome
Source: Sci Rep. 2019 Nov 27;9:17677. doi: 10.1038/s41598-019-53623-2 (PMC6881409; doi:10.1038/s41598-019-53623-2)
Supplement: Supplementary file 1 — Supplementary Information [file 41598_2019_53623_MOESM1_ESM.docx]

*Supplementary Information*

**A Plant Growth-Promoting Microbial Soil Amendment Dynamically Alters the Strawberry Root Bacterial Microbiome**

**Siwen Deng^1,2*^, Heidi M.-L. Wipf^1,2*^, Grady Pierroz^1,2^, Ted K. Raab^3^, Rajnish Khanna^4^, Devin Coleman-Derr^1,2^**

^1^Department of Plant and Microbial Biology, University of California, Berkeley, CA, USA

^2^Plant Gene Expression Center, USDA-ARS, Albany, CA, USA

^3^Carnegie Institution for Science, Department of Plant Biology, Stanford, CA, USA

^4^i-Cultiver, Inc., 404 Clipper Cove Way, San Francisco, CA, USA

**Author for correspondence:**

Devin Coleman-Derr

Tel: 1-510-559-5911

Email: [colemanderr@berkeley.edu](mailto:colemanderr@berkeley.edu)

**^*^**Indicates first authors

**1 Supplementary Figures and Tables**

**1.1 Supplementary Figures**

**Supplementary Figure S1.** Experimental design. Overview of location and field setup. Map (Important Farmland in California, 2014 © California Department of Conservation, Division of Land Resource Protection, 2017. FMMP Survey Cycle Years 2012-2014 Data, Map Published January 2017.) showing farm location (red star) in which study was (34.9716° N, 120.5718° W). Amendment-treated (colored blue) and control (yellow) strawberry plants were planted in neighboring 12-acre plots. At each time point, plants were chosen at random from control and amendment-treated plots, where six replicates of bulk soil (taken 6 inches away from the plant), rhizosphere, and root samples were collected for microbial community assessment.


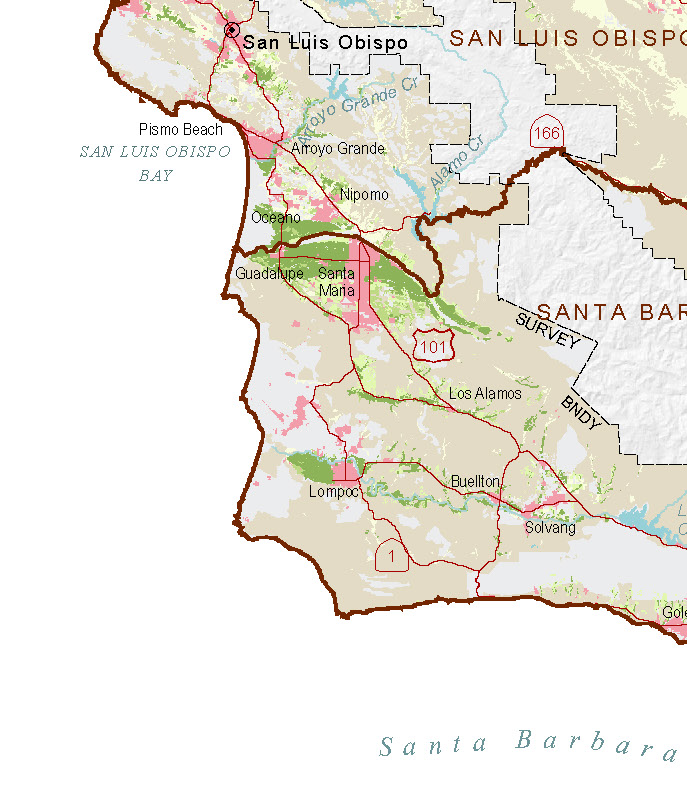


Treated

Control

Root

Rhizosphere

Soil

Treated

Control


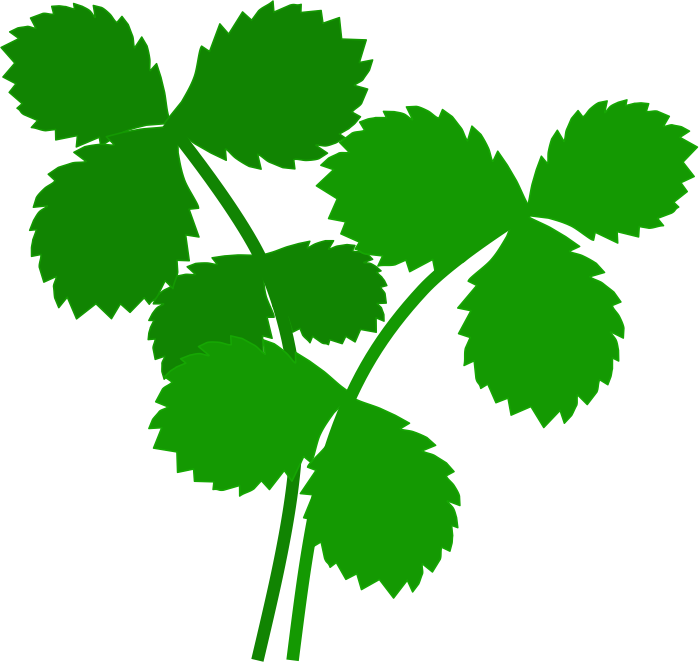

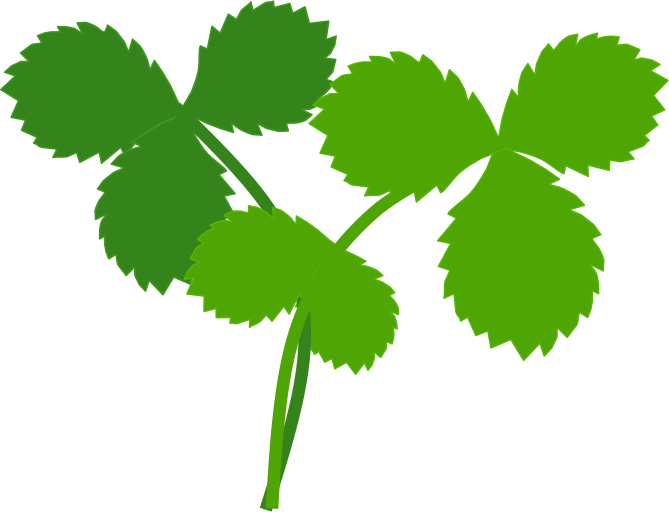


Prime Farmland

Unique Farmland

Grazing Land

Farmland of State-

wide Importance

Farmland of

Local Important

Urban and Built-

Up Land

Other Land

Water

**Root Dry Weight (g)**

3

2

1

**Time Point**

1^st^

2^nd^

3^rd^

Control

Treated

Treatment:

**Supplementary Figure S2.** Treatment effect on strawberry plant dry weight. Line plot illustrating the average trend of 5 to 6 replicates per treatment per time point for root dry weight across the three sampling time points.

4000

3000

2000

2000

0

0

50000

100000

150000

6

4

2

0

0

50000

100000

150000

Control Soil

Treated Soil

Control Rhizosphere

Treated Rhizosphere

Control Root

Treated Root

**Sequencing depth**

**Shannon’s Diversity Index**

**Number of Observed Species**

**(a)**

**(b)**


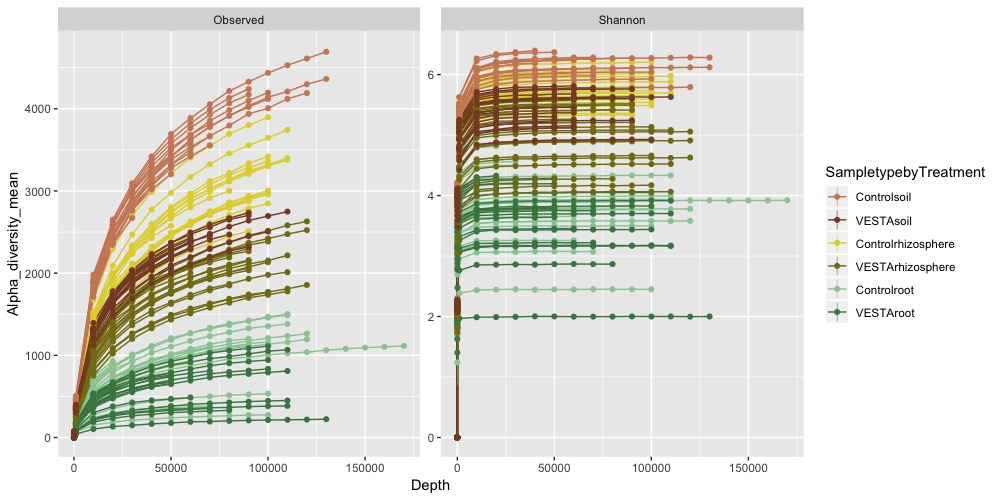

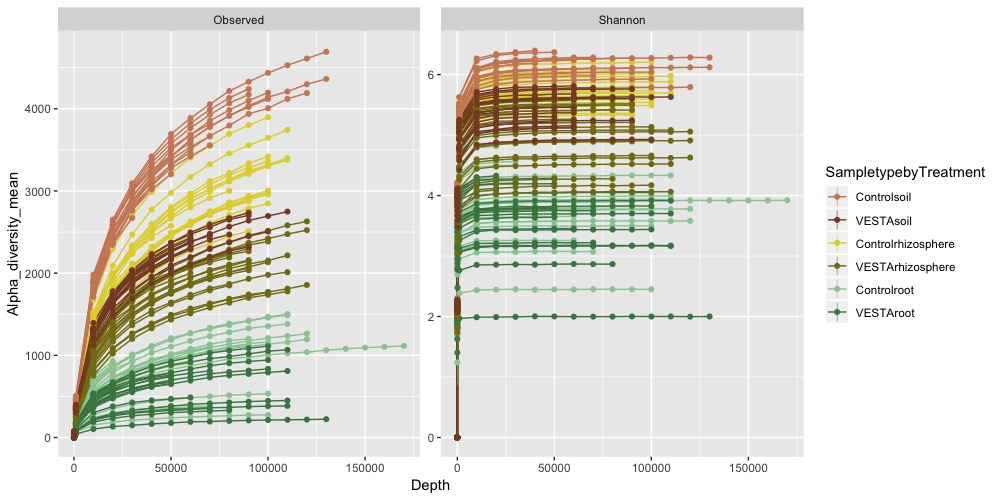


**Supplementary Figure S3.** Rarefaction curves to estimate alpha diversity, (a) Shannon’s diversity indices, and (b) number of observed species, as a function of sequencing depth.

**(a)**

Soil

Rhizosphere

Root

**(b)**

**PCoA 2 (14.4%)**

**PCoA 1 (52.6%)**

-0.2

0.0

0.2

0.1

0.0

-0.1

-0.1

0.1

1^st^

2^nd^

3^rd^

4^th^

Control

Treated

**Number of Observed Species**

Soil

Rhizosphere

Root

1^th^

2^nd^

3^rd^

4^th^

1600

1400

1200

1500

1250

1000

600

400

200

**Treatment**

Control

Treated

Control

Treated

Control

Treated

Control

Treated

**Supplementary Figure S4.** Amendment application correlates to reduced numbers of observed species, and samples cluster by treatment. (a) Box-and-whisker plots of the numbers of observed species in amendment-treated (blue) and control (yellow) samples for each sample type (soil, rhizosphere, root) and across the four sampling time points (1^st^, 2^nd^, 3^rd^, 4^th^). The horizontal line within each box represents the median. The bottom and top edges of each box indicate the 25^th^ and 75^th^ percentiles, respectively. Individual points are outliers. (b) Principal coordinate analysis (PCoA) plot for all samples generated based on weighted UniFrac distances. The first two axes explain 67.0% of the data, with the primary axis (52.6% of variance) primarily distinguishing samples by sample type and the secondary axis (14.4% of variance) distinguishing samples by treatment type (amendment-treated or control).

**Relative Abundance**

1.00

0.75

0.50

0.25

0.00

Other

Gemmatimonadetes

Deltaproteobacteria

Flavobacteria

Bacilli

Acidobacteria

Actinobacteria

TM7PH

SR1PH

Betaproteobacteria

Gammaproteobacteria

Sphingobacteria

Alphaproteobacteria

**(a)**

**(b)**

Pre-product 1

Pre-product 2

Week 0

Week 1

Week 2

Week 3

Week 4

Week 5

Week 6

Week7

Week 8

Week 9

Week 11

Week 12

Week 13

Week 0

Week 1

Week 2

Week 3

Week 4

Week 5

Week 6

Week 7

Week 8

Week 9

Week 11

Week 12

Week 13

Pre-product 1

Pre-product 2

Week0

Week1

Week2

Week3

Week4

Week5

Week6

Week7

Week8

Week9

Week11

Week12

Week13

0.75

0.50

0.25

0.00

Distance

Pre-product 1

Pre-product 2

**Supplementary Figure S5.** Bacterial communities shift from the pre-products throughout a 13 weeks time course of the amendment. (a) Relative abundance bar graphs of the bacterial classes present in the initial pre-product 1 BHF-10^®^ and pre-product 2 SOBEC^®^, as well as samples of the product VESTA^®^ collected over a time course of 13 weeks. Week 0 was collected immediately after mixing the two pre-products, Week 1 was collected after one week, Week 2 was collected after two weeks, etc. (b) Heat map representative of the pairwise Bray-Curtis distances between samples of the two pre-products and the samples of the amendment product across the 13 week time course the time points of which the amendment product was sampled. Degree of blue shading corresponds to Bray-Curtis distance value, where darker shading corresponds to less distance between the bacterial community profiles of samples.

Other

Unclassified

Gemmatimonadetes

Deltaproteobacteria

Flavobacteria

Cytophagia

Clostridia

Bacilli

Acidobacteria

Actinobacteria

Betaproteobacteria

Gammaproteobacteria

Sphingobacteria

Alphaproteobacteria

**Relative Abundance**

1.00

0.75

0.50

0.25

0.00

16S data

Shotgun data

**(a)**

**(b)**

**Supplementary Figure S6.** Shotgun metagenomic analysis of the amendment product. (a) Comparison of the relative abundances of bacterial classes between 16S rRNA amplicon sequencing and shotgun whole genome sequencing of the amendment product. This slightly differs from 16S rRNA bacterial relative abundances. (b) Functional profile of the amendment product and the relative abundances of genes ascribed to the listed categories, where relative abundances range from 0.1% - 14.5% and greater enrichment is indicated by corresponding block being more darkly shaded. There is high enrichment in carbohydrate, amino acid, and protein metabolism.

313

831

192

35

172

85

1141

63

78

21

18

3

3

8

20

Rhizosphere

Root

Soil

Amendment

Soil

Rhizosphere

Root

Amendment

**Supplementary Figure S7.** A majority of OTUs in the amendment product (as shown by the blue sphere) are present either within the soil (brown), rhizosphere (yellow), or root (green) compartment of treated samples. Venn-Diagram displaying the numbers of OTUs from amendment-treated soil, rhizosphere, and root samples that are shared or unique to the OTUs found in the amendment. OTUs used as determined by 16S amplicon sequencing.

**1.2 Supplementary Tables**

**Supplementary Table 1.** Nutrient analysis of the product VESTA. The nutrient profile of the liquid product VESTA assessed by D&D Agricultural Laboratory, Inc. (Fresno, CA) by means of a pH analyzer, electrical conductivity meter for soluble salts, FP-528 (Leco Corporation, St. Joseph, MI) for total nitrogen, and Optima 8000 ICP-OES (PerkinElmer, Inc., Waltham, MA) for all other parameters.

| Parameter | Amount |
| --- | --- |
| pH | 6.89 |
| Soluble Salts (Electrical Conductivity) | 0.61 dS/m |
| Total Nitrogen | 0.002 % |
| Phosphorus (P) | 0.017 |
| Diphosphorus Pentoxide (P_2_O_5_) | 0.039 % |
| Potassium (K) | 0.099 % |
| Potassium Oxide (K_2_O) | 0.119 % |
| Calcium Carbonate (CaCO_3_) | 0.010 % |
| Magnesium Carbonate (MgCO_3_) | 0.003 % |
| Zinc | 0.49 ppm |
| Manganese | 1.05 ppm |
| Iron | 172.0 ppm |
| Copper | 0.84 ppm |
| Boron | 3.17 ppm |
| Sodium | 0.002 % |
| Chloride | 0.004 % |

**Supplementary Table 2.** Field soil chemical analyses show nutrient levels and other soil parameters differ in soils treated with the product in comparison to controls. (a) Soil pH was measured with 2.00 grams of soil with a Fischer Tris-electrode after a 3-point calibration (n=13 control; m=9 treated). Electrical conductivity was also measured from 2 grams of water solubilized soil with a Thermo-Russell RL060-C conductivity meter, and total nitrogen and carbon were measured with a CarloErba1500N elemental analyzer. Total nitrogen and carbon data reported here is the average of two replicates. Nitrate levels were determined by WESCOR analyses on n=11 soil samples from control, and m=10 from the amendment-treated fields. Exchangeable Ca analyzed over 3 time points, mean + standard error of mean for n=10 controls, m=9 treated. (b) Total elemental composition of soils, as determined by energy-dispersive X-ray fluorescence (EDXRF); values are expressed as mean ± standard error of the mean in dry weight or µg/g dry wt, and n=5 controls, m=4 for amendment-treated plants. Though many other elements were determined, their values were near instrumental detection limits, as EDXRF is only suitable for elements between Z> 11 (Na) and Z<92 (U). MANOVA significance values when tested for differences between values for treated and control samples: * (P<0.05), ** (P<0.01), and *** (P<0.001).

| a. Chemical parameters measured in soil from treated and control fields in Guadeloupe, CA. | | |
| --- | --- | --- |
| Parameter | Amount in Control Soil | Amount in Treated Soil |
| pH | 7.32 + 0.04 | 7.44 + 0.04 |
| Electrical Conductivity | 184 + 15 µS/cm | 242 + 22 µS/cm * |
| Total Nitrogen | 0.177% dry weight | 0.188% dry weight |
| Total Carbon | 0.249% dry weight | 0.385% dry weight |
| Nitrate | 23.2 + 1.51 ppm | 18.6 + 1.51 ppm * |
| Ammonium | 6.36 + 0.96 ppm | 5.80 + 0.93 ppm |
| Exchangeable [Ca^++^] | 1256 + 34 mg/kg | 1427 + 23 mg/kg *** |
| b. Total elemental composition of soils from treated and control fields after strawberry growth reached stage T_4_. | | |
| Parameter | Amount in Control Soil | Amount in Treated Soil |
| pH | 7.32 + 0.04 | 7.44 + 0.04 |
| Electrical Conductivity | 184 + 15 µS/cm | 242 + 22 µS/cm * |
| Total Nitrogen | 0.177% dry weight | 0.188% dry weight |
| Total Carbon | 0.249% dry weight | 0.385% dry weight |
| Nitrate | 23.2 + 1.51 ppm | 18.6 + 1.51 ppm * |
| Ammonium | 6.36 + 0.96 ppm | 5.80 + 0.93 ppm |
| Exchangeable [Ca^++^] | 1256 + 34 mg/kg | 1427 + 23 mg/kg *** |

**Supplementary Table 3.** Chemical analyses of treated and untreated strawberry plants from time points 1 through 4 show amendment-treated plants differ in the amount of various elements in roots and leaves. (a) Acid-digestible nutrient pools from strawberry root tissues collected at time points 1-3, as determined by inductively coupled plasma emission spectroscopy (ICP-EOS); values are expressed as mean ± standard error of the mean in µg/g dry wt, and n=8, m=8 for control and treated plants. (b) Total elemental composition by EDXRF of strawberry foliage; values are composited from n=5 controls, m=5 for VESTA-treated plants. NBS-1533a Standard pine needles were also run on the same instrument, and elements for which certificate values are registered were satisfactorily quantified/recovered. MANOVA significance values when tested for differences between values for control and treated samples: * (P<0.05), ** (P<0.01), and *** (P<0.001).

| a. Acid-digestible nutrient pools from strawberry root tissues of plants grown in treated and control fields, collected at time points 1-3. | | |
| --- | --- | --- |
| Nutrient | Amount in Control Roots (µg/g) | Amount in Treated Roots (µg/g) |
| Aluminum | 3114 ± 371 | 2073 ± 172 * |
| Boron | 25.65 ± 8.61 | 15.41 ± 6.71 |
| Calcium | 8760 ± 692 | 7068 ± 521 |
| Potassium | 4006 ± 374 | 4511 ± 294 |
| Magnesium | 3916 ± 229 | 3684 ± 283 |
| Phosphorus | 1747 ± 107 | 2315 ± 89.6 *** |
| Silicon | 511.4 ± 70.3 | 447.3 ± 71.8 |
| Sulfur | 18496 ± 3029 | 16192 ± 1282 |
| Iron | 2995 ± 340 | 1951 ± 151 * |
| Copper | 33.9 ± 14.9 | 19.96 ± 1.81 |
| Manganese | 103.6 ± 12.1 | 105.6 ± 9.90 |
| Molybdenum | 3.505 ± 0.288 | 2.602 ± 0.193 * |
| Sodium | 1096 ± 159 | 1415 ± 191 |
| Zinc | 53.62 ± 9.37 | 52.44 ± 2.65 |
| b. Total elemental composition of strawberry foliage from plants grown in treated and control fields, collected at time point 4. | | |
| Nutrient | Amount in Control Leaves | Amount in Treated Leaves |
| Sodium | 0.313 % | 0.386 % |
| Magnesium | 0.249 % | 0.292 % |
| Aluminum | < 0.0020 % | < 0.0020 % |
| Silicon | 0.129 % | 0.274 % |
| Phosphorous | 0.177% | 0.251 % |
| Sulfur | 0.119 % | 0.168 % |
| Chlorine | 0.07025 % | 0.109 % |
| Potassium | 0.739 % | 0.992 % |
| Calcium | 0.466% | 0.688 % |
| Titanium | 18.5 mg/kg | 27.5 mg/kg |
| Vanadium | 0.2 mg/kg | 0.7 mg/kg |
| Chromium | 0.6 mg/kg | 0.8 mg/kg |
| Manganese | 34.9 mg/kg | 52.3 mg/kg |
| Iron | 177.6 mg/kg | 283.1 mg/kg |
| Cobalt | < 3.0 mg/kg | < 2.9 mg/kg |
| Nickel | 6.7 mg/kg | 9.4 mg/kg |
| Molybdenum | 6.1 mg/kg | 3.4 mg/kg |

**Supplementary Table 4.** Sample type, treatment, and time point are significant factors explaining the variation in Shannon’s Diversity for all samples. Rhizospheres and soils are significantly different between amendment-treated and control samples of the same compartment. (a) Results from a Tukey's HSD (honest significant difference) test of Shannon's Diversity indices, as based on Bray-Curtis distances. ‘SD’ = standard deviation. (b) ANOVA analyses for Shannon's Diversity across all samples based on the mixed linear model with factors sample type, treatment, time point and replicate as explanatory variables. Significant factors indicated by ***, denoting a p value of less than .001.

| a. Results from a Tukey's HSD (honest significant difference) test of Shannon's Diversity indices, as based on Bray-Curtis distances. | | | | | | | |
| --- | --- | --- | --- | --- | --- | --- | --- |
| SampleType by Treatment | Means | S.D. | R | Min | | Max | Groups |
| Soil.Control | 5.7984 | 0.1464 | 19 | 5.5038 | | 6.0671 | a |
| Rhizosphere.Control | 5.5786 | 0.2135 | 24 | 5.1865 | | 5.9477 | ab |
| Soil.Treated | 5.2411 | 0.2392 | 20 | 4.7793 | | 5.5888 | bc |
| Rhizosphere.Treated | 4.9072 | 0.4673 | 23 | 3.9585 | | 5.4575 | c |
| Root.Control | 3.8148 | 0.5585 | 24 | 2.4223 | | 4.9298 | d |
| Root.Treated | 3.6779 | 0.5394 | 24 | 1.9864 | | 4.2547 | d |
| b. ANOVA analyses for Shannon's Diversity across all samples based on the mixed linear model with factors sample type, treatment, time point and replicate as explanatory variables. | | | | | | | |
| Factor | DF | Sum of Squares | Mean Squares | F Statistics | p-value | |  |
| SampleType | 2 | 82.59 | 41.30 | 271.701 | <0.001 | | *** |
| Treatment | 1 | 6.68 | 6.68 | 43.968 | <0.001 | | *** |
| Time point | 3 | 4.20 | 1.40 | 9.202 | <0.001 | | *** |
| Replicate | 5 | 0.32 | 0.06 | 0.417 | 0.836 | |  |
| Residuals | 122 | 18.54 | 0.15 |  |  | |  |

**Supplementary Table 5.** Table of Kruskal-Wallis test results comparing Shannon indices between treated and untreated samples within each sample type and time point. Significant factors indicated by *, **, and ***, denoting p value of less than .05, .01, and .001, respectively.

| Sample Type | Time Point | Kruskal-Wallis Test | |  |
| --- | --- | --- | --- | --- |
|  |  | chi-squared | p-value |  |
| Soil | 1 | 8.308 | 0.0039 | ** |
| Soil | 2 | NA | NA |  |
| Soil | 3 | 8.308 | 0.0039 | ** |
| Soil | 4 | 8.308 | 0.0039 | ** |
| Rhizosphere | 1 | 7.410 | 0.0065 | ** |
| Rhizosphere | 2 | 8.308 | 0.0039 | ** |
| Rhizosphere | 3 | 8.308 | 0.0039 | ** |
| Rhizosphere | 4 | 6.533 | 0.0106 | * |
| Root | 1 | 1.256 | 0.2623 |  |
| Root | 2 | 1.256 | 0.2623 |  |
| Root | 3 | 0.641 | 0.4233 |  |
| Root | 4 | 5.026 | 0.0250 | * |

**Supplementary Table 6.** Treatment and time point are highly significant factors explaining the variance in bacterial community differences between samples within each compartment (soil, rhizosphere, and root). Table of PERMANOVA analysis results using Bray-Curtis distances and performed independently on each sample type using the adonis function within the package vegan in R [^70^](https://paperpile.com/c/UaENUc/R6S5). ‘TRT” = treatment factor, ‘DF’ = degrees of freedom, and significant factors indicated by *, **, and ***, denoting p value of less than .05, .01, and .001, respectively.

| Sample Type | Factors | DF | Sum of Squares | Mean Squares | F Statistic | R^2^ | p-value |  |
| --- | --- | --- | --- | --- | --- | --- | --- | --- |
| Soil | Treatment | 1 | 1.4555 | 1.4555 | 35.278 | 0.4226 | <0.001 | *** |
| Soil | Time point | 3 | 0.4781 | 0.1594 | 3.863 | 0.1388 | <0.001 | *** |
| Soil | TRT : Time point | 3 | 0.2314 | 0.0772 | 1.870 | 0.0672 | 0.0286 | * |
| Soil | Residuals | 31 | 1.2790 | 0.0413 | 0.371 |  |  |  |
| Soil | Total | 38 | 3.4440 |  |  | 1.0000 |  |  |
| Rhizosphere | Treatment | 1 | 1.8962 | 1.8962 | 31.429 | 0.3164 | <0.001 | *** |
| Rhizosphere | Time point | 3 | 1.2491 | 0.4164 | 6.901 | 0.2084 | <0.001 | *** |
| Rhizosphere | TRT : Time point | 3 | 0.4942 | 0.1647 | 2.731 | 0.0825 | <0.001 | *** |
| Rhizosphere | Residuals | 39 | 2.3530 | 0.0603 | 0.393 |  |  |  |
| Rhizosphere | Total | 46 | 5.9926 |  |  | 1.0000 |  |  |
| Root | Treatment | 1 | 3.4715 | 3.4715 | 45.377 | 0.3924 | <0.001 | *** |
| Root | Time point | 3 | 1.6196 | 0.5399 | 7.057 | 0.1831 | <0.001 | *** |
| Root | TRT : Time point | 3 | 0.6952 | 0.2317 | 3.029 | 0.0786 | 0.0014 | ** |
| Root | Residuals | 40 | 3.0601 | 0.0765 | 0.346 |  |  |  |
| Root | Total | 47 | 8.8464 |  |  | 1.0000 |  |  |

**Supplementary Table 7.** Sample type, treatment, and time point are highly significant factors explaining the variance in bacterial community differences between samples. (a) Table displaying results of a canonical analysis of principal coordinates (CAPS) for all samples testing the hypothesis that sample type, treatment, time point, and replicate are significant factors explaining the variation between all samples in either Bray-Curtis or weighted UniFrac distances. (b) Table displaying results of CAPS done for samples in each sample type (soil, rhizosphere, and root) individually, to test the hypothesis that treatment, time point, and replicate are significant factors in explaining the variation between samples in either Bray-Curtis or weighted UniFrac distances. ‘DF’ = degrees of freedom, and significant factors indicated by ***, denoting p value of less than .001.

| a. Results from CAPS using Bray-Curtis and UniFrac distances to determine significant factors explaining variance across bacterial communities. | | | | | | |
| --- | --- | --- | --- | --- | --- | --- |
| Factors | Distance Metric | DF | Sum of Squares | F Statistics | p-value |  |
| Sample Type | UniFrac | 2 | 2.8844 | 108.4102 | 0.001 | *** |
| Treatment | UniFrac | 1 | 0.5436 | 40.8648 | 0.001 | *** |
| Time point | UniFrac | 3 | 0.2954 | 7.4008 | 0.001 | *** |
| Replicate | UniFrac | 5 | 0.0652 | 0.9804 | 0.447 |  |
| Residual | UniFrac | 122 | 1.6230 |  |  |  |
| Sample Type | Bray-Curtis | 2 | 13.5589 | 68.5415 | 0.001 |  |
| Treatment | Bray-Curtis | 1 | 4.0932 | 41.3828 | 0.001 | *** |
| Time point | Bray-Curtis | 3 | 1.6578 | 5.5868 | 0.001 | *** |
| Replicate | Bray-Curtis | 5 | 0.4650 | 0.9402 | 0.548 | *** |
| Residual | Bray-Curtis | 122 | 12.0670 |  |  |  |

| b. Results from CAPS using Bray-Curtis and UniFrac distances done separately for each sample type. | | | | | | | |
| --- | --- | --- | --- | --- | --- | --- | --- |
| Sample Type | Distance Metric | Factors | DF | Sum of Squares | F Statistic | p-value | % Variance |
| Soil | UniFrac | Treatment | 1 | 0.1171 | 34.8504 | 0.001 | 0.43 |
| Soil | UniFrac | Time point | 3 | 0.0443 | 4.3922 | 0.001 | 0.16 |
| Soil | UniFrac | Replicate | 5 | 0.0166 | 0.9847 | 0.487 | 0.06 |
| Soil | UniFrac | Residual | 29 | 0.0975 | 0.2755 | 0.354 |  |
| Soil | Bray | Treatment | 1 | 1.4555 | 32.6404 | 0.001 | 0.42 |
| Soil | Bray | Time point | 3 | 0.4781 | 3.5739 | 0.001 | 0.14 |
| Soil | Bray | Replicate | 5 | 0.2173 | 0.9746 | 0.453 | 0.06 |
| Soil | Bray | Residual | 29 | 1.2931 | 3.4440 | 0.375 |  |
| Rhizosphere | UniFrac | Treatment | 1 | 0.1971 | 24.2593 | 0.001 | 0.26 |
| Rhizosphere | UniFrac | Time point | 3 | 0.2171 | 8.9069 | 0.001 | 0.29 |
| Rhizosphere | UniFrac | Replicate | 5 | 0.0322 | 0.7930 | 0.759 | 0.04 |
| Rhizosphere | UniFrac | Residual | 37 | 0.3007 | 0.7472 | 0.402 |  |
| Rhizosphere | Bray | Treatment | 1 | 1.8962 | 27.6167 | 0.001 | 0.32 |
| Rhizosphere | Bray | Time point | 3 | 1.2491 | 6.0640 | 0.001 | 0.21 |
| Rhizosphere | Bray | Replicate | 5 | 0.3068 | 0.8936 | 0.635 | 0.05 |
| Rhizosphere | Bray | Residual | 37 | 2.5405 | 5.9926 | 0.424 |  |
| Root | UniFrac | Treatment | 1 | 0.5103 | 33.9420 | 0.001 | 0.34 |
| Root | UniFrac | Time point | 3 | 0.3451 | 7.6526 | 0.001 | 0.23 |
| Root | UniFrac | Replicate | 5 | 0.0779 | 1.0361 | 0.419 | 0.05 |
| Root | UniFrac | Residual | 38 | 0.5713 | 1.5045 | 0.380 |  |
| Root | Bray | Treatment | 1 | 3.4715 | 39.2592 | 0.001 | 0.39 |
| Root | Bray | Time point | 3 | 1.6196 | 6.1052 | 0.001 | 0.18 |
| Root | Bray | Replicate | 5 | 0.3951 | 0.8937 | 0.613 | 0.04 |
| Root | Bray | Residual | 38 | 3.3602 | 8.8464 | 0.380 |  |

**Supplementary Table 8.** Several members of the kingdom Bacteria, phylum Proteobacteria, and class Betaproteobacteria that are more enriched in treated root samples have potential functions of sulfur cycling, denitrification, and nitrogen fixation. Summary of read counts and potential activities of Betaproteobacteria genera that are enriched in root samples with amendment treatment with potential function predicted based on a literature search. ‘RC’ = number of read counts.

| OTU  Rank | Order | Family | Genus | p-value | Control  RC | Treated  RC | Potential Function |
| --- | --- | --- | --- | --- | --- | --- | --- |
| 13 | *Burkholderiales* | *Comamonadaceae* | *Acidovorax* | 0.001 | 2261 | 16523 | Sulfur cycling |
| 11 | *Burkholderiales* | *Burkholderiales* | *unclassified* | 0.003 | 1007 | 14086 | . |
| 15 | *Methylophilales* | *Methylophilaceae* | *Methylotenera* | 0.008 | 4930 | 8628 | De- nitrification |
| 20 | *Methylophilales* | *Methylophilaceae* | *Methylotenera* | 0.001 | 878 | 8547 | De- nitrification |
| 22 | *Burkholderiales* | *Comamonadaceae* | *Comamonadaceae* | 0.001 | 768 | 3966 | Sulfur cycling |
| 55 | *Methylophilales* | *Methylophilaceae* | *Methylophilaceae* | 0.001 | 569 | 2454 | De- nitrification |
| 158 | *Burkholderiales* | *Comamonadaceae* | *Ramlibacter* | 0.001 | 20 | 232 | Nitrogen fixation |
| 465 | *Methylophilales* | *Methylophilaceae* | *Methylophilaceae* | 0.001 | 8 | 126 | De- nitrification |
| 479 | *Burkholderiales* | *Comamonadaceae* | *Comamonadaceae* | 0.001 | 1 | 103 | Sulfur cycling |
| 261 | *Rhodocyclales* | *Rhodocyclales* | *Rhodocyclales* | 0.008 | 16 | 79 | Nitrogen fixation |
| 96 | *Rhodocyclales* | *Rhodocyclales* | *Rhodocyclales* | 0.007 | 18 | 70 | Nitrogen fixation |
| 1054 | *Rhodocyclales* | *Rhodocyclaceae* | *Methyloversatilis* | 0.001 | 2 | 55 | . |
| 1703 | *Burkholderiales* | *Burkholderiaceae* | *Chitinimonas* | 0.003 | 0 | 24 | . |
| 233 | *Neisseriales* | *Neisseriaceae* | *Vogesella* | 0.004 | 0 | 20 | . |
| 412 | *Burkholderiales* | *Comamonadaceae* | *Comamonadaceae* | 0.007 | 1 | 16 | Sulfur cycling |
